# Introductory Remarks

**Published:** 1881-10

**Authors:** Samuel Wilkes


### Selections.

### INTRODUCTORY REMARKS.

### DELIVERED UPON THE OPENING OF THE SECTION ON PATHOLOGY.

By Samuel Wilkes, M. D., F. R. S.

Our subject, in a word, is Pathology. Pathology has received various definitions, the most common being that which contrasts it with Physiology; for as the latter is regarded as the science of healthy organic life, so the former has been held to be the science of the unhealthy or of the abnormal course of life contrasted with the norm l. This division of vital action into normal and abnormal is true in a superficial sense, and might be made theoretically to stand as a definition, but it is by no means applicable to our practical science of pathology, nor can it be made of any value as an expression of diagnostic knowledge in treating the thousand ills to which flesh is heir.

In the first place, it must be admitted that the changes which occur in every organic structure, as years roll on, are to be regarded as normal, unless we take an imaginary or ideal standard of a being living in some former golden age, where nought was known but perpetual youth, and regard every departure from this as morbid. Although we do not frame such a picture to ourselves, but know that the various changes in the bones, the cartilages, the lungs, the brain, and other parts which take place in age are in harmony with the dictates of Nature, yet how often are we called upon to treat these changes as forms of disease? They are, however, no more unnatural or pathological than the sere and yellow leaf which falls from the oak in autumn.

If, however, these senile changes occur prematurely, they will then be abnormal, and may be strictly regarded as morbid. Herein is one form of a pathological condition with which we have to deal—a premature decay arising from the various causes which bring the organism to an end, either from their operating with unusual force or from some inherent weakness in the body, which is unable to moderate their action. Now, if all these potent influences, instead of driving the mechanism too quickly, and so bringing it prematurely to an end, concentrate their forces upon one organ only, that organ would become, in ordinary parlance, diseased; but the process there set up may be of exactly the same nature as time would otherwise have produced. In comparatively young persons, for instance we meet with fibroid and fatty changes in the heart and vessels, distention of the air-cells, alterations in the structure of bones and joints, which resemble in every respect those which age would have ordinarily induced. Therefore many of the conditions which we call disease seem nothing more than the result of the concentration on a particular organ of all those agencies which, under ordinary circumstances, bring about senile changes. These changes, therefore, although senile in character, are abnormal, and therefore may be rightly regarded as pathological.

The pathologist, therefore, cannot but regard the body in the first place in its physiological relations with its surroundings, and mark the alterations which time produces. The physiologist is aware that the production of force must be accompanied by loss elsewhere, seeing that gain and loss are equal, and therefore in observing organic life he must regard the destructive processes as well as the formative. Life seems to depend upon changes continually going on in relation with the atmosphere in which all living bodies are steeped. The burning of the fuel in oxygen supplies the forces necessary for living processes; we therefore, although alive, are constantly being consumed. During so many years the body is undergoing combustion, or, we might say, slow destruction, and this process occurs much more rapidly in some persons and in some animals than in others. Why one creature should live longer or burn out sooner than another is not clear; why, for example, should a dog be worn out in ten or twelve years, its limbs be stiff, its sight and hearing impaired, its intelect obtuse, and senile changes be discoverable in its brain and elsewhere, when a parrot may take a century for the production of the same destructive changes? Why tissues of the same composition should wear out in one animal after ten revolutions of the earth when it takes a hundred revolutions to destroy similar ones in another, is by no means apparent. In man, if the destructive and reproductive changes are normally counterbalanced, the ordinary duration of life is reached. If the balance be not kept, the destructive agencies may be in the ascendency, and life be shortened. If any of the ordinary surroundings which are always exerting their influences upon us, as various kinds of air, food, moral and mental moods, be in any way noxious, they may in time tend to premature death; and if they should act in such a manner as to cause localized organic changes, we should style these changes disease. There can be little doubt that a large number of maladies in England, as gout, Bright's disease, etc., are induced by mere excesses or inequalities in a mode of life which is considered ordinarily correct. It ought to be one of our studies to consider the relations of the human race to the soil, and observe all the circumstances which centuries have induced to bring about this normal or healthy relation between them. We might then observe the effects of the concentration of some of the more untoward of the influences which ordinarily environ it, as well as inquire into the effects of transplantation into another country. It seems that all the usual surroundings of life in civilized society, acting in undue proportion or in a more determined manner, induce a very large number of the diseases which we are called upon to treat.

In considering all these agencies working for what we call evil, and leading to destruction, we must not overlook an opposing law—that of reparation. Not only do we observe a production of living force in necessary association with a dissolution of material, but an ever-existing tendency towards the remaking of the injured tissues. We can scarcely think of a morbid change in the body which is not attended by another which has an opposite tendency. Every phthisical lung showing destruction of the tissue exhibits at the same time the attempt to limit the process and to save life by shutting off the escape of air from the lung or sealing the ulcerated blood-vessels.

Then, again, in considering the definition of disease, after having observed how large a number of maladies are produced by the influences of all our ordinary surroundings, we have to recognize those external causes of an extraordinary or specific character which prey upon the human frame, and often bring its machinery to an end. Now, if these causes are obviously parasitic, we are not witnessing so much the case of disease as the spectacle of one animal praying upon another. As regards the parasite, it is pursuing its normal life history, and as regards the patient or the host, he is simply being destroyed; the difference in his mode of death from that which would result from the onslaught of a wild animal would consist merely in time. If a man fall a victim to the bite of a cobra, he is not said to die of disease; but the term is applicable if he die of glanders. There is this difference, however, in the latter case—the poison is not a natural one even in the infecting animal. If, however, in these infectious diseases the morbific cause be an animal or vegetable organism, although microscopic, then we really have to deal with the operation of one living being acting upon another, and the so-called specific malady exhibits nothing more than the natural course of life of certain specific organisms. The term disease, according to the definition, is here again scarcely applicable.

All these abnormalities of the human organism, under whatever conditions they may arise, suggest that as every branch of biological science is being studied in relation to the lower organizations, and according to the law of evolution, so must pathology become the subject of a large field of inquiry, and be made to embrace the diseases of all animal and vegetable life. The comparison of disease in man and animals may throw much light upon its nature, and it is remarkable that so few persons have been stimulated to the work, by considering the long controversy which has taken place as to the relation between vaccina and variola, or hydrophobia and rabies. A true human pathology should have its basis in comparative pathology. Here lies a mine of wealth but little worked. As at the present time every structure and function of the human body is being studied in reference to its antecedents in the lower animals, so there can be no doubt that the various morbid changes to which it is liable may be also profitably discussed in reference to similar actions in more simple forms of life. The truth of this has been clearly seen by philosophers who have had no special acquaintance with our department of science. Thus Buckle, in his History of Civilization in England, says: "The best Physiologists distinctly recognize that the basis of their science must include not only the animals below man, but also the entire vegetable kingdom, and that without this commanding survey of the whole realm of organic nature we cannot possibly understand even human physiology, still less general physiology. The Pathologists, on the other hand, are so much in arrear, that the diseases of the lower animals rarely form parts of their plan, while the diseases of plants are almost entirely neglected, although it is certain that until all these have been studied, and some steps taken to generalize them, every pathological condition will be eminently empirical on account of the narrowness of the field from which it is collected." This is almost as true now as when written, several years ago; but we are pleased to think that our countryman, Sir James Paget, has already removed this slur upon our scientific procedure by his lecture on "Elemental Pathology," in which he shows the importance of observing the resemblances between the changes in the various tissues of man and the vegetable world, and also the deductions to be drawn therefrom.

Again, if the specific diseases be due to organisms, and the hypothetical *contagium vivum* be a reality, it must be subject to the same laws as other organic matter; and if the doctrine of evolution be true, it must have numerous relations with families of its own kind, and perhaps with others which are now obsolete. This idea has occupied the minds of several medical men in this country, and it will no doubt further fructify in their hands.

A highly contagious disease prevailing in a particular locality may be exhibiting the differentiation of some more simple, less virulent, and wide-spread disorder. For example, a slightly contagious epidemic sore-throat might in course of time develop into a more virulent one until it culminated in diphtheria; and if this disease be due to an organism, the latter might have found a more genial soil for its development, or be altered by propagation and time, so that new properties might at last have been added to it. There may be a progressive development of infectiveness. Then, again, the doctrine of natural selection might obtain in the fact of some specific diseases remaining amongst us, while others have become obsolete. The same law, too, if allowed its full operation, might tend still more than it does to the subjugation of many hereditary diseases; for as these appear in youth, and often cause death, they would fade away by a process of self-destruction. As regards the specific diseases, we see again how the most susceptible persons would be struck out by the poison and the least susceptible remain, so that the poison would be modified in its virulence. We witness this fact in the more moderate characters of the exanthemata in all civilized nations, in comparison with the more profound effects produced by them in nations where the diseases had been hitherto unknown, as, for example, the fatality in the Pacific Islands of our comparatively mild British measles.

Besides the maladies which are induced by the evil influences of our ordinary surroundings, and those due to specific causes just named, there is a class of diseases styled new-growths, which take a very large share in adding to man's mortality. The advance made in our knowledge of these structures is very considerable, and is still rapidly progressing towards a determination of their origin and the discovery of their relation to the normal tissues. These investigations are assisting us in discarding some of our older notions regarding their constitutional and malignant nature, and proving that many are accidental in their origin, and therefore may possibly be averted.

In these brief remarks we see how the simple definition of pathology as a deviation from the healthy standard fails in its application, and how wide is the range of subjects included in its domain. What these are, you, gentlemen, are about to illustrate in the different subjects which you will bring before the section.—*Boston Medical and Surgical Journal.*

